# Fifty years of the Revista de Saúde Pública

**DOI:** 10.1590/S01518-8787.2016050006855ED

**Published:** 2016-02-19

**Authors:** Carlos Augusto Monteiro, Rita Barradas Barata, José Leopoldo Ferreira Antunes

**Affiliations:** I Faculdade de Saúde Pública. Universidade de São Paulo. São Paulo, SP, Brasil; II Faculdade de Ciências Médicas. Santa Casa de São Paulo. São Paulo, SP, Brasil

In 2016, the *Revista de Saúde Pública* launches its 50^th^ volume and celebrates 50 years of uninterrupted existence. Half a century ago, an exceptional group of masters – Armando Piovesan, Elza Salvatori Berquó, Flavio Wagner Rodrigues, Oswaldo Paulo Forattini, Paulo Nogami, Reinaldo Ramos, and Walter Belda – accepted the challenge proposed by the former Director of the Faculdade de Saúde Pública of the Universidade de São Paulo, Rodolfo dos Santos Mascarenhas, to create a new scientific journal that met the international standards of scientific rigor and independence.

The goal would be to encourage and promote the production of knowledge in Public Health, especially of the most relevant knowledge, to improve the health conditions of the Brazilian population. The strategy was to offer the producers and users of this knowledge a vehicle not restricted to the scientific production of the School itself.

In these 50 years, the *Revista de Saúde Pública* has not deviated from these guidelines. During all these years, many difficulties have been overcome (Antunes et al., 2015). The journal grew, improved, and became more modern, reaffirming its commitment to the health of the Brazilian population. In accordance with the prospect of internationalization, the journal is indexed in the main bibliographic databases, such as PubMed Central (PMC), PubMed, Web of Science, Embase, Scopus, LILACS, among others. From 1967 to 2015, over 4,000 articles were published – all available online at SciELO.

The main health problems that affect the Brazilian population, studied under different angles and multiple analytical approaches, are constantly present in the pages of the *Revista de Saúde Pública.* Throughout its existence, the journal has followed the evolution of the agenda of priorities of Public Health in Brazil, the success and failure of public health policies, the emergence of new themes, health surveillance, planning and provision of health services, as well as the universalization of the population access to these services. The journal has also followed the demographic, socioeconomic, political, and cultural changes that occurred in the Country and the development of scientific disciplines that support research in Public Health.

Reviewing the scientific literature published by the *Revista de Saúde Pública*, it has occurred to us to invite experts from different areas of Public Health to review the main issues that have been mentioned in its pages, identifying how problems and analytical approaches have evolved over the decades in each thematic area. Our expectation is for these articles to be published in 2016. We also expect to offer, at the end of the year, a seminar to celebrate the 50 years of the *Revista de Saúde Pública*, so these articles can be presented by their authors and discussed in person among colleagues.

To conclude, we would like to thank everyone who took part in the journal’s history, its readers, authors, reviewers, editors, and management support staff. On behalf of everyone, we pay our homage to the previous scientific editors, who led the editorial work and taught us so much about this activity. To Professor Oswaldo Paulo Forattini, who was its scientific editor for four decades, promoted the journal both nationally and internationally, being a great source of inspiration for those who followed his steps. Later, we could count on Professors Moisés Goldbaum and Aluísio Jardim Dornellas de Barros in the scientific editorial office. Their performance was also very important to promote the journal in the scientific, academic, and sanitary fields. The *Revista de Saúde Pública* had the privilege of having Professor Maria Teresinha Dias de Andrade as its Executive Editor throughout all of these 50 years. Without her, it would have been impossible to maintain and enhance the enviable editorial quality of the journal. As this vehicle’s current scientific editors, and certainly on behalf of authors, reviewers, readers, and editors of the *Revista de Saúde Pública*, we have a lot to thank these people for!
