# Supplementary material for: Fifty years of the Revista de Saúde Pública
Source: Rev Saude Publica. 2016 Feb 19;50:1. doi: 10.1590/S01518-8787.2016050006855ED (PMC4772693; doi:10.1590/S01518-8787.2016050006855ED)
Supplement: Translation

# Translation: Cinquenta anos da Revista de Saúde Pública

**Authors:** Carlos Augusto Monteiro, Rita Barradas Barata, José Leopoldo Ferreira Antunes

**Affiliations:** I Faculdade de Saúde Pública. Universidade de São Paulo. São Paulo, SP, Brasil; II Faculdade de Ciências Médicas. Santa Casa de São Paulo. São Paulo, SP, Brasil

Em 2016, a *Revista de Saúde Pública* lança seu quinquagésimo volume e comemora cinquenta anos de existência ininterrupta. Há meio século, um grupo excepcional de mestres – Armando Piovesan, Elza Salvatori Berquó, Flavio Wagner Rodrigues, Oswaldo Paulo Forattini, Paulo Nogami, Reinaldo Ramos e Walter Belda – aceitou o desafio proposto pelo então Diretor da Faculdade de Saúde Pública da Universidade de São Paulo – Rodolfo dos Santos Mascarenhas – para criar uma nova revista científica, que atendesse aos padrões internacionais de rigor científico e independência.

A meta seria estimular e divulgar a produção do conhecimento em Saúde Pública, em particular do conhecimento mais relevante para a melhoria das condições de saúde da população brasileira. A estratégia era oferecer aos produtores e usuários desse conhecimento um veículo que não fosse restrito à produção científica da própria Faculdade.

Nestes cinquenta anos, a *Revista de Saúde Pública* não se afastou dessas diretrizes. Durante todos estes anos, muitas dificuldades foram superadas (Antunes et al., 2015). A Revista cresceu, se aprimorou e se modernizou, reafirmando seu compromisso com a saúde do povo brasileiro. Consoante com a perspectiva de internacionalização, a Revista está indexada nas principais bases bibliográficas, como PubMed Central (PMC), PubMed, Web of Science, Embase, Scopus, Lilacs, dentre outras. De 1967 a 2015 foram publicados mais de 4.000 artigos – todos disponíveis *online* no SciELO.

Os principais problemas de saúde que afetam a população brasileira, estudados sob diferentes ângulos e múltiplas abordagens analíticas, são presença constante nas páginas da *Revista de Saúde Pública*. Ao longo de sua existência, a Revista tem acompanhado a evolução da agenda de prioridades em Saúde Pública no Brasil, o sucesso e o insucesso das políticas públicas de saúde, a emergência de novos temas, a vigilância em saúde, o planejamento e a provisão dos serviços de saúde, bem como a universalização do acesso da população a esses serviços. A Revista também tem acompanhado as mudanças demográficas, socioeconômicas, políticas e culturais ocorridas no País e o desenvolvimento das disciplinas científicas que amparam a pesquisa em Saúde Pública.

Revendo o conjunto da produção científica divulgada pela *Revista de Saúde Pública*, ocorreu-nos convidar especialistas de diferentes áreas da Saúde Pública para revisarem os principais temas que têm sido referidos em suas páginas, identificando como os problemas e as abordagens analíticas evoluíram ao longo das décadas em cada área temática. Nossa expectativa é que esses artigos sejam publicados em 2016. Contamos, ainda, em realizar, no final do ano, um seminário comemorativo dos cinquenta anos da *Revista de Saúde Pública*, no qual esses trabalhos possam ser apresentados por seus autores e discutidos presencialmente com os colegas.

Para concluir, gostaríamos de registrar nosso agradecimento especial a todas as pessoas que fazem a história da Revista, seus leitores, autores, revisores, editores e pessoal de apoio gerencial. Representando a todos, rendemos nossa homenagem aos editores científicos anteriores, que conduziram o trabalho editorial e muito nos ensinaram sobre essa atividade. O Professor Oswaldo Paulo Forattini, que foi seu Editor Científico por quatro décadas, projetou a Revista nacional e internacionalmente, sendo grande fonte de inspiração para os que lhe sucederam. Posteriormente, pudemos contar com os Professores Moisés Goldbaum e Aluísio Jardim Dornellas de Barros na editoria científica. Sua atuação também foi preponderante para projetar a Revista no cenário científico, acadêmico e sanitário. A *Revista de Saúde Pública* tem o privilégio de contar com a Professora Maria Teresinha Dias de Andrade como Editora Executiva ao longo de todos os seus cinquenta anos. Sem ela, teria sido impossível manter e aperfeiçoar a invejável qualidade editorial da *Revista de Saúde Pública*. Como atuais editores científicos deste veículo, e certamente expressando os autores, revisores, leitores e editores da *Revista de Saúde Pública,* temos muito a agradecer a essas pessoas!
